# β-Tocotrienol Decreases PDGF-BB-Induced Proliferation and Migration of Human Airway Smooth Muscle Cells by Inhibiting RhoA and Reducing ROS Production

**DOI:** 10.3390/ph17060712

**Published:** 2024-05-30

**Authors:** Aditya Sri Listyoko, Ryota Okazaki, Tomoya Harada, Miki Takata, Masato Morita, Hiroki Ishikawa, Yoshihiro Funaki, Akira Yamasaki

**Affiliations:** Division of Respiratory Medicine and Rheumatology, Department of Multidisciplinary Internal Medicine, Faculty of Medicine, Tottori University, Yonago 683-8504, Japan; okazaki0222@tottori-u.ac.jp (R.O.); tomo.h.308@tottori-u.ac.jp (T.H.); mikiyamamu@tottori-u.ac.jp (M.T.); drmasato@tottori-u.ac.jp (M.M.); h.ishikawa1990@tottori-u.ac.jp (H.I.); yfunaki@tottori-u.ac.jp (Y.F.)

**Keywords:** airway remodeling, ASM, asthma, Rho-A, ROS, tocotrienol, vitamin E

## Abstract

Background: Tocotrienols exhibit antioxidant and anti-inflammatory activities. RhoA, a small GTPase protein, plays a crucial role in regulating contractility in airway smooth muscle (ASM). Previous studies have demonstrated that γ-tocotrienols reduce ASM proliferation and migration by inhibiting the activation of RhoA. In this present study, we investigate the effect of another vitamin E isoform, β-tocotrienols, on human ASM cell proliferation and migration stimulated by platelet-derived growth factor-BB (PDGF-BB). Methods: Human ASM cells were pre-treated with β-tocotrienol prior to being stimulated with PDGF-BB to induce ASM cell proliferation and migration. The proliferation and migration of PDGF-BB-induced human ASM cells were assessed using colorimetric and transwell migration assays. The intracellular ROS assay kit was employed to quantify reactive oxygen species (ROS) in human ASM cells. Additionally, we explored the effect of β-tocotrienols on the signaling pathways involved in PDGF-BB-induced ASM proliferation and migration. Results: β-tocotrienol inhibited PDGF-BB-induced ASM cell proliferation and migration by reducing RhoA activation and ROS production. However, in this present study, β-tocotrienol did not affect the signaling pathways associated with cyclin D1, phosphorylated Akt1, and ERK1/2. Conclusions: In conclusion, the inhibition of RhoA activation and ROS production by β-tocotrienol, resulting in the reduction in human ASM proliferation and migration, suggests its potential as a treatment for asthma airway remodeling.

## 1. Introduction

Asthma typically presents as a heterogeneous disease, often marked by chronic inflammation of the airways [1]. Persistent chronic inflammation in asthma may lead to structural alterations known as airway remodeling [2,3]. Airway remodeling in asthma encompasses a spectrum of structural alterations, including changes such as epithelial alterations including epithelial–mesenchymal transition [4], sub-epithelial fibrosis including a fibroblast-to-myofibroblast transition and deposition of extracellular matrix [5,6], alterations in airway smooth muscle (ASM) [7,8], and remodeling of the airway vasculature [9]. Airway remodeling may be associated with poor asthma outcomes, potentially leading to increased airway hyperresponsiveness and decreased lung function [10,11,12].

Numerous studies have explored the association between vitamins and asthma, suggesting the potential benefits of supplementary vitamin intake in asthma management to improve outcomes, particularly for individuals deficient in vitamin levels [13,14,15,16]. The biological role of vitamin E metabolites has garnered considerable attention; however, exploration of the role of vitamin E metabolites in respiratory diseases remains notably limited. Eight isoforms of vitamin E have been discovered to date, including α, β, γ, and δ-tocotrienol and α, β, γ, and δ-tocopherol [17,18]. Several studies have noted no significant difference in antioxidant capacity between tocopherol and tocotrienol [19,20], emphasizing the importance of considering experimental conditions for accurate comparisons. However, other studies have highlighted the higher potential antioxidant activity in α-tocotrienol compared to α-tocopherol [21] and the potential benefits specifically associated with tocotrienols including antiangiogenic [22], anticancer [23,24], cerebral, and cardioprotective effects [25,26], a potential therapeutic effect on diabetes and hyperlipidemia [27,28,29], and anti-inflammatory effects [30,31].

In the context of lung disease, despite limited studies, tocotrienols have been observed to provide benefits. For instance, protective effects of γ-tocotrienol against emphysema and lung function were observed in a chronic obstructive pulmonary disease mouse model [32]. Another study observed that tocotrienol has the potential to ameliorate pulmonary fibrosis through mechanisms involving the transforming growth factor-β1 (TGF-β1)/Smad, phosphatidylinositol 3′-kinase/Akt (PI3K/Akt), and translocations of nuclear factor-kappa B (NF-κB) signaling pathways [33]. In the context of asthma, particularly airway remodeling, tocotrienols may offer benefits. Our previous study revealed that γ-tocotrienols could potentially influence airway remodeling by inhibiting RhoA activation in platelet-derived growth factor-BB (PDGF-BB)-induced ASM cell proliferation and migration [34]. Another study determined that γ-tocotrienols hold potential benefits in modulating airway remodeling by inhibiting RhoA activation in TGF-β-stimulated ASM cells [35].

Several studies have compared the antioxidant effects of tocotrienols with inconsistent results. One study, employing peroxide and thiobarbituric acid as markers, assessed similar levels of antioxidant capacity between β-tocopherol and β-tocotrienol, while α-tocotrienol and α-tocopherol were less potent. Moreover, γ-tocotrienol, δ-tocopherol, and δ-tocotrienol exhibited slightly higher potency than either β-tocopherol or β-tocotrienol [36]. Another study observed that administration of a tocotrienol-rich fraction (TRF), containing α-tocotrienol, β-tocotrienol, δ-tocotrienol, γ-tocotrienol, and α-tocopherol, exhibited similar effects on antioxidant levels compared to α-tocopherol alone [37]. Regarding effectiveness as an anticancer treatment, β-tocotrienol showed a notably stronger antiproliferative effect compared to γ-tocotrienols in human breast adenocarcinoma cell lines MDA-MB-231 and MCF7 [38]. Given this, the variability in potency among isoforms of vitamin E can be attributed to several factors. These factors include the source of vitamin E utilized, research methodologies employed, and the intended purpose of administering this isoform, whether as an antioxidant, anticancer agent, or for other potential uses.

Our previous study focused on the use of γ-tocotrienols on ASM cells. However, the effects of various isoforms of tocotrienols as potential therapies for airway remodeling, derived from vitamin E, have not been thoroughly investigated and remain incompletely understood. Furthermore, there have been no studies comparing different isoforms of vitamin E as potential treatments for airway remodeling. In this present study, we investigate the effect of another vitamin E isoform, β-tocotrienols, on human ASM cell proliferation and migration stimulated by platelet-derived growth factor-BB (PDGF-BB). Additionally, we explored the effect of β-tocotrienols on the signaling pathways involved in PDGF-BB-induced ASM proliferation and migration.

## 2. Results

### 2.1. β-Tocotrienols Inhibit PDGF-BB-Induced ASM Cell Proliferation

**Figure 1:**
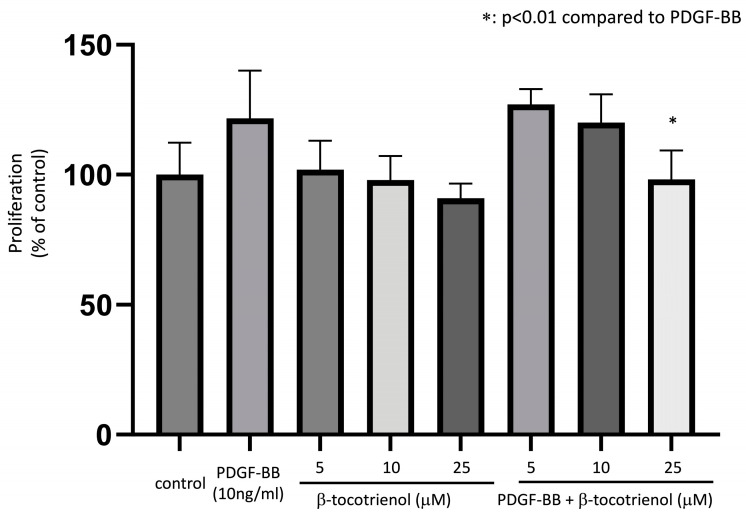
Effect of β-tocotrienols on ASM cell proliferation. Human ASM cells were cultured with 10 ng/mL PDGF-BB, 5–25 μM β-tocotrienols, or pre-treated with 5–25 μM β-tocotrienols before 10 ng/mL PDGF-BB stimulation. The proliferation of ASM cells was measured using the Cell Counting Kit (CCK-8). The results are presented as a percentage of the non-stimulated (control) group and expressed as the mean ± standard deviation (SD) of at least three independent experiments (*: *p* < 0.01 compared to PDGF-BB stimulation alone).

Human ASM cells were pre-treated with various concentrations of β-tocotrienols for 1 h before being stimulated with 10 ng/mL PDGF-BB for 48 h to induce ASM cell proliferation. PDGF-BB stimulation increased ASM cell proliferation compared to the non-stimulated (control) group. Pre-treatment with 25 μM β-tocotrienols significantly reduced this effect (Figure 1), indicating that β-tocotrienols have an inhibitory effect on PDGF-BB-induced ASM cell proliferation.

### 2.2. β-Tocotrienols Inhibit PDGF-BB-Induced ASM Cell Migration

**Figure 2:**
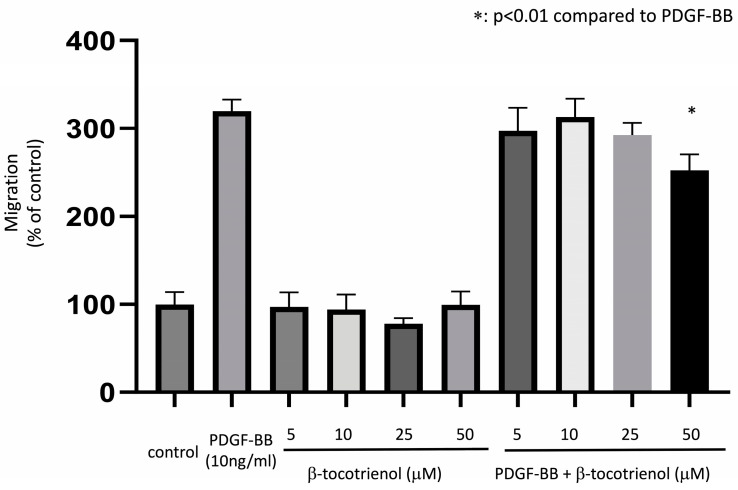
Effect of β-tocotrienols on ASM cell migration. Human ASM cells were cultured with 10 ng/mL PDGF-BB, 5–50 μM β-tocotrienols, or pre-treated with 5–50 μM β-tocotrienols before 10 ng/mL PDGF-BB stimulation. The migration of ASM cells was evaluated using a transwell chamber. The results are presented as a percentage of the non-stimulated (control) group and expressed as the mean ± standard deviation (SD) of at least three independent experiments (*: *p* < 0.01 compared to PDGF-BB stimulation alone).

Human ASM cells were pre-treated with various concentrations of β-tocotrienols for 1 h before being stimulated with 10 ng/mL PDGF-BB for 5 h to induce ASM cell migration. The migration of ASM cells increased in the PDGF-BB group compared to the non-stimulated (control) group. Pre-treatment with 50 μM β-tocotrienols significantly reduced this effect (Figure 2), indicating the inhibitory effect of β-tocotrienols on PDGF-BB-induced ASM cell migration.

### 2.3. β-Tocotrienols Reduce Intracellular ROS Production

**Figure 3:**
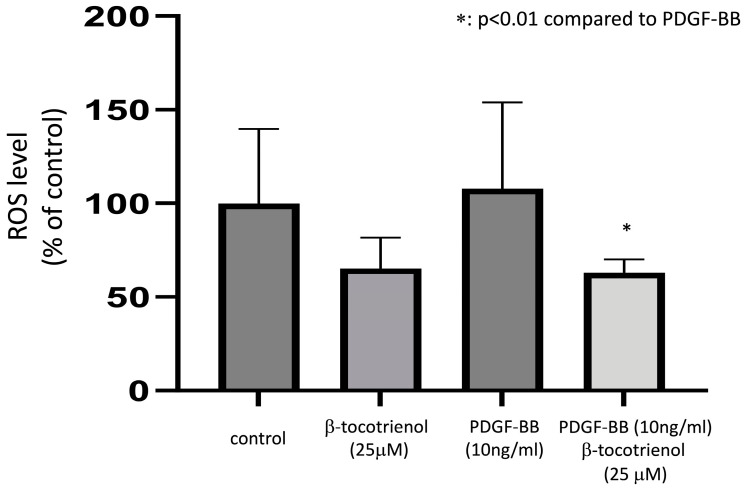
Effect of β-tocotrienols on ROS production. Human ASM cells were cultured with 10 ng/mL PDGF-BB, 25 μM β-tocotrienols, or pre-treated with 25 μM β-tocotrienols before 10 ng/mL PDGF-BB stimulation. The level of ROS in ASM cells was quantified after stimulation with 10 ng/mL PDGF-BB using the Oxiselect intracellular ROS assay kit. The results are presented as a percentage of the non-stimulated (control) group and expressed as the mean ± standard deviation (SD) of at least three independent experiments (*: *p* < 0.01 compared to PDGF-BB stimulation alone).

The generation of oxygen species is essential for the mitogen stimulation of ASM, initiating signal transduction pathways that lead to cell proliferation [39]. In this current study, we assessed the impact of β-tocotrienols on reactive oxygen species (ROS) production. The ROS production decreased after stimulation with β-tocotrienols compared to the non-stimulated (control) group, although the result did not reach statistical significance. Following the stimulation with PDGF-BB, ROS production increased, and pre-treatment with β-tocotrienols ameliorated this effect (Figure 3), indicating the inhibitory effect of β-tocotrienols on ROS production.

### 2.4. The Effect of β-Tocotrienols on Cyclin D1 Levels

**Figure 4:**
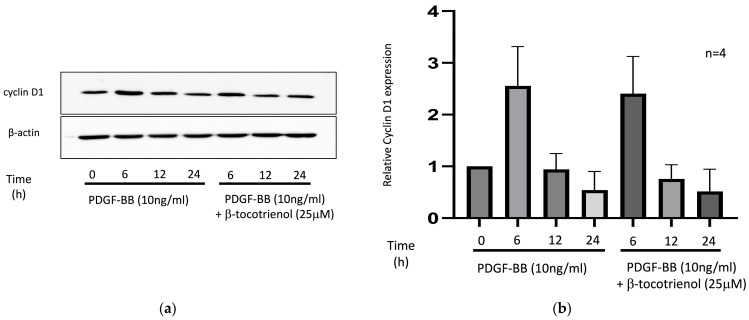
Effect of β-tocotrienols on cyclin D1 expression: (**a**) human ASM cells were cultured with 10 ng/mL PDGF-BB or pre-treated with 25 μM β-tocotrienols before 10 ng/mL PDGF-BB stimulation. Western blot was used to evaluate the expression of cyclin D1. (**b**) The graphic represents densitometry calculations of relative protein expression/β-actin. The results are presented as the mean ± standard deviation (SD) of at least three independent experiments.

Cyclin D1 serves as a crucial regulator of the cell cycle, including that of ASM cells [40,41,42]. The previous study observed that γ-tocotrienol reduced PDGF-BB-induced cyclin D1 expression [34]. The cyclin D1 level exhibited an upregulation after stimulation with PDGF-BB for 6 h. However, pre-treatment with β-tocotrienols did not reduce this effect (Figure 4), indicating that cyclin D1 may not be associated with the effect of β-tocotrienols on inhibiting PDGF-BB-induced ASM cell proliferation and migration.

### 2.5. The Effect of β-Tocotrienols on Akt1 and ERK1/2 Signaling Pathways

**Figure 5:**
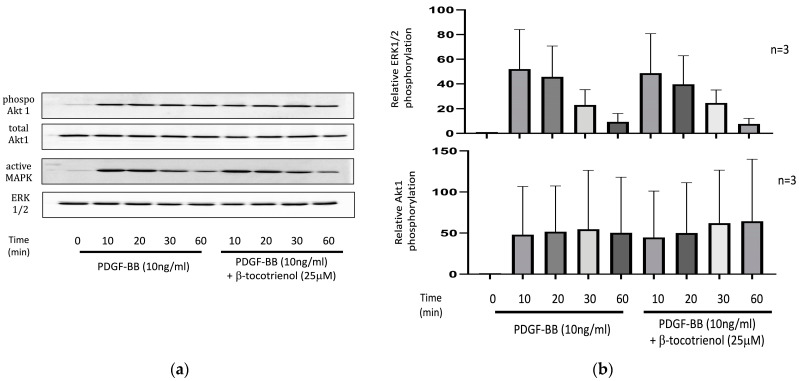
Effect of β-tocotrienols on Akt1 and ERK1/2 phosphorylation: (**a**) Human ASM cells were cultured with 10 ng/mL PDGF-BB or pre-treated with 25 μM β-tocotrienols before 10 ng/mL PDGF-BB stimulation. Western blot was used to evaluate the expression of Akt1 and ERK1/2. (**b**) The graphic represents densitometry calculations of phosphorylated protein targets to total protein. The results are presented as the mean ± standard deviation (SD) of at least three independent experiments.

The previous study observed that the proliferation and migration inhibition effect of γ-tocotrienol on ASM cells was not associated with the Akt1 and ERK1/2 signaling pathways [34]. In this present study, we assessed the effect of β-tocotrienols on the Akt1 and ERK1/2 signaling pathways associated with PDGF-BB-induced proliferation and migration. Phosphorylation of Akt1 and extracellular signal-regulated kinase1/2 (ERK1/2) increased following PDGF-BB stimulation. Pre-treatment with β-tocotrienols did not inhibit this effect (Figure 5), indicating that phosphorylation of Akt1 and ERK1/2 may not be associated with the effect of β-tocotrienols on ASM cell proliferation and migration.

### 2.6. β-Tocotrienols Inhibit PDGF-BB-Induced ASM Cell Proliferation and Migration via RhoA Inactivation

**Figure 6:**
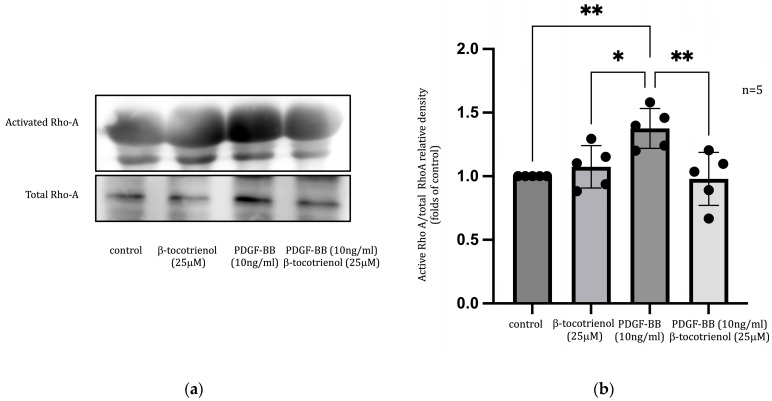
Effect of β-tocotrienols on RhoA activity: (**a**) human ASM cells were cultured with 10 ng/mL PDGF-BB or pre-treated with 25 μM β-tocotrienols before 10 ng/mL PDGF-BB stimulation. Rho activity was evaluated using a Rho pull-down assay, and the expression was determined with Western blot analysis. (**b**) The graphic represents densitometry calculations of active RhoA to total RhoA. The data are presented as the mean ± standard deviation (SD) of at least three independent experiments. (*: *p* < 0.05, **: *p* < 0.01, compared to PDGF-BB stimulation).

The Rho family serves as a crucial regulator of various cell functions, including migration, adhesion, proliferation, and differentiation [43,44,45]. The previous study observed that γ-tocotrienol inhibited PDGF-BB-induced RhoA activation [34]. In this current study, we observed that activation of RhoA increased following PDGF-BB stimulation, and pre-treatment with β-tocotrienols reduced this effect (Figure 6), indicating that the inhibition effect of β-tocotrienols on ASM cell proliferation and migration is associated with the RhoA pathway.

## 3. Discussion

**Figure 7:**
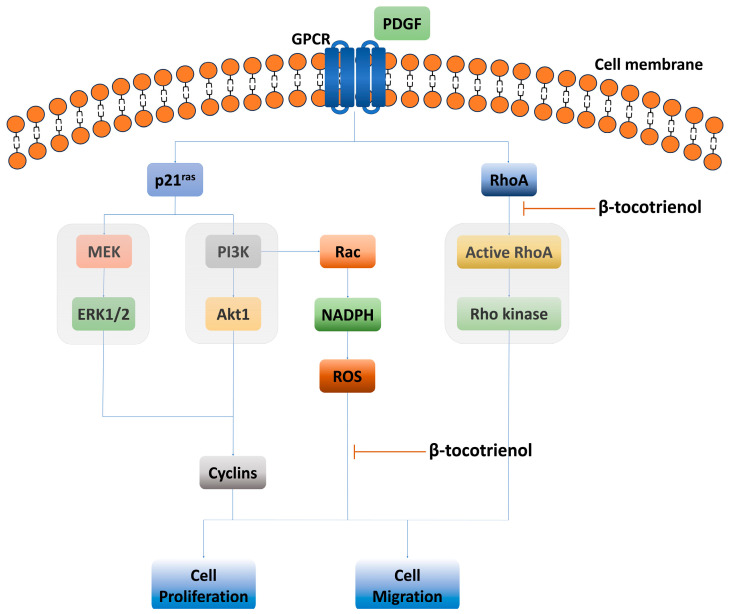
Schematic representation of the effects of β-tocotrienol on inhibiting ASM proliferation and migration. β-tocotrienol directly inhibits ROS production and induces RhoA inactivation. However, β-tocotrienol may not inhibit the Akt1, ERK, and cyclin signaling pathways. Platelet-derived growth factor-BB (PDGF-BB), G protein-coupled receptor (GPCR), extracellular signal-regulated kinase (ERK), phosphatidylinositol 3′-kinase (PI3K), reactive oxygen species (ROS), and nicotinamide adenine dinucleotide phosphate (NADPH).

In this current study, we observed that the proliferation and migration of PDGF-BB-induced ASM cells were effectively inhibited by β-tocotrienol (Figure 7). These recent findings with β-tocotrienol are consistent with our previous study, where γ-tocotrienol inhibited PDGF-BB-induced ASM cell proliferation and migration [34]. Given that ASM proliferation and migration are key contributors to airway remodeling, playing a pivotal role in both promoting and mediating the remodeling process [46,47], these results support the hypothesis that tocotrienols, both of β-tocotrienol and γ-tocotrienol, may hold potential as a treatment for airway remodeling in asthmatic patients.

The mechanism underlying ASM proliferation, migration, growth, and remodeling has been the subject of various studies and literature reviews [7,48,49,50,51,52]. Our findings underscore the importance of targeting the Rho pathway, particularly with tocotrienols, as both β-tocotrienol and γ-tocotrienol inhibit RhoA activation related to ASM proliferation and migration, suggesting that the Rho pathway is an important pathway inhibited by vitamin E isoforms to prevent ASM remodeling [34]. This is supported by several studies and reviews indicating that the Rho/ROCK pathway is a critical regulator of airway remodeling, influencing the regulation, proliferation, and contraction of ASM cells [48,53,54,55]. The Rho family of GTPases is a group of small signaling G proteins, forming a subfamily within the Ras superfamily and comprising six isoforms (A, B, C, D, E, and G). RhoA has been extensively studied and demonstrates both GDP and GTP binding activities. When stimulated by G protein-coupled receptor (GPCR) agonists, the inactive GDP-bound RhoA can convert into its active state, GTP-bound RhoA [56]. Interestingly, the administration of a selective Rho kinase inhibitor attenuates airway inflammation, eosinophilia, and airway hyperresponsiveness [57,58,59]. Another study observed that the administration of a selective Rho-A/Rho kinase inhibitor resulted in a significant reduction in inflammatory cell count, decreased mucous secretion, and lowered expression of MUC5AC. These effects were associated with the downregulation of interleukin-17 (IL-17), IL-4, and IL-13 levels, as well as a decline in the expression and phosphorylation of NFκB and signal transducer and activator of transcription 6 (STAT6) [60], indicating the potential therapeutic targeting of the Rho/Rho kinase downstream pathway in asthma. 

In this present study, β-tocotrienol did not inhibit the phosphorylation of cyclin D1, Akt1, and ERK 1/2 in PDGF-BB-induced ASM cell proliferation and migration, suggesting that these pathways might not be associated with the inhibitory effect of β-tocotrienol on ASM cell proliferation and migration. Our previous study observed that although γ-tocotrienol also did not affect the Akt1 and ERK 1/2 signaling pathways, cyclin D1 was associated with the inhibition of ASM proliferation and migration. This suggests that various isoforms of vitamin E may have different mechanisms associated with the inhibition of ASM remodeling. ASM proliferation involves crosstalk between inflammatory mediators, contractile agonists, and growth factors released during inflammation. This condition triggers ASM proliferation through activation of the MAPKs, PI3K/AKT, and JAK2/STAT3 signaling pathways. The migration of ASM cells is also a multifaceted process, with several signaling molecules, such as FAK, PI3K, PTEN, ERK, p38, Src, Rho kinase, c-Abl, and Wnt/β-catenin, identified as contributors to the regulation of ASM cell migration [52]. Exploring both upstream and downstream ASM proliferation and migration signaling pathways may provide clear insight into the precise mechanism by which tocotrienol inhibits ASM proliferation and migration.

The reduction in ROS production by β-tocotrienol was observed in this current study. This result is in line with our previous study, which observed a decrease in ROS levels in the γ-tocotrienol pre-treatment group compared to the PDGF-BB group. Several studies have observed the influence of oxygen species generation in ASM proliferation [39,61,62], highlighting the importance of targeting ROS in airway remodeling. The precise mechanism of the antioxidant activity of β-tocotrienol is not fully understood, and further studies are needed to elucidate this aspect. However, several potential mechanisms can be considered, including the enhancement of antioxidant enzymes including glutathione peroxidase activity, scavenging of free radicals, and inhibition of lipid peroxidation [63,64]. The comparative antioxidant strength of β-tocotrienol to other isoforms of vitamin E remains unclear. Nevertheless, tocotrienols, in general, have been suggested to exhibit more potent antioxidant activity than tocopherol [63,64,65,66].

In this study, we did not assess the anti-inflammatory effects of β-tocotrienol, and further investigations are required to understand the precise mechanism of β-tocotrienol as an anti-inflammatory agent. β-tocotrienol has the potential to exert a therapeutic effect on airway remodeling by diminishing ASM proliferation and migration. Furthermore, it may possess anti-inflammatory properties, thereby contributing to the amelioration of airway remodeling in asthma. The anti-inflammatory effects of tocotrienol have been observed in several studies. For instance, the reduction in several pro-inflammatory cytokines, including tumor necrosis factor-α (TNF-α), IL-4, and IL-8, through the inhibition of inducible NO synthase (iNOS) and COX-2 expression, was observed after administration of a tocotrienol-rich fraction [31]. Another study observed a reduction in mRNA and protein expressions of TNF-α, IL-1β, IL-6, and iNOS via inhibition of the nuclear translocations of NF-κB and activator protein-1 (AP-1) after treatments with δ-tocotrienol [30], suggesting that tocotrienol has potent anti-inflammatory properties that may be beneficial in airway inflammation and remodeling.

In this study, we did not assess the comparative effects between β-tocotrienol and γ-tocotrienol or with other isoforms of vitamin E. Comparative studies between vitamin E isoforms are limited to antioxidant comparisons between tocotrienol and tocopherol isoforms [36,37], or comparisons in the antiproliferative effects in cancer cells [38]. For instance, comparisons between β-tocotrienol and γ-tocotrienol demonstrated a notably stronger anti-proliferative effect in β-tocotrienol in human breast adenocarcinoma cell lines MDA-MB-231 and MCF7 [38]. This study may offer insight that β-tocotrienol has a more potent cytotoxic effect compared to γ-tocotrienol, suggesting that using β-tocotrienol instead of γ-tocotrienol to target normal cells should be considered for this effect. In this study, we did not conduct viability or apoptotic tests in normal human ASM cells. However, our findings indicated that the administration of 5–25 μM β-tocotrienols alone did not significantly decrease ASM cell proliferation and compared to the non-stimulated group. This suggests that at this dose, β-tocotrienols do not exert a cytotoxic effect on human ASM cell lines.

Clinical studies on Vitamin E administration in asthma have not yet yielded conclusive results to date. Asthmatic patients have been shown to exhibit lower dietary vitamin E levels [67], with severe asthma patients exhibiting lower vitamin E levels compared to those with mild asthma [13]. Additionally, a different study observed that supplemental vitamin E during pregnancy could prevent asthmatic diseases [68]. In the context of clinical studies involving vitamin E isoform, γ-tocopherol administration significantly decreased induced sputum neutrophils compared to the placebo [69], indicating the potential benefit of the vitamin E isoform in reducing neutrophilic airway inflammation. However, one study found that vitamin E supplementation did not affect asthma control in mild to moderate adult asthma subjects [70].

Further investigations are warranted to elucidate the potential benefits of vitamin E and its isoforms for the clinical management of asthma and airway remodeling. Assessing the effects of anti-inflammatory-related vitamin E isoforms should be a priority. Evaluating other signaling molecules or pathways that may indirectly influence ASM remodeling may provide clear insight into the precise mechanism by which tocotrienol inhibits ASM proliferation and migration. Evaluating the effects of other vitamin E isoforms, such as α- or δ-tocotrienol, is also important. Assessing the effects of vitamin E isoforms on other cells, such as airway epithelial cells, may offer benefits as airway remodeling involves not only ASM cells. While the in vitro findings are promising, future studies should validate the efficacy of vitamin E isoform in relevant animal models of asthma to ascertain their therapeutic potential in vivo, including investigating the long-term effects of tocotrienols on ASM remodeling in animal models of asthma or exploring synergistic effects with existing asthma medications, which would enrich the discussion and stimulate further scientific inquiry.

## 4. Materials and Methods

### 4.1. Human ASM Cells and Culture Conditions

Immortalized human ASM cells, achieved through stable expression of human telomerase reverse transcriptase (hTERT), were used in this present experiment. These cells were generously provided by Dr. Andrew J. Halayko from the University of Manitoba. The cells were cultured in Dulbecco’s Modified Eagle’s Medium (DMEM) supplemented with 10% Fetal Bovine Serum (FBS), 100 μg/mL penicillin, and 100 μg/mL streptomycin then incubated at 37 °C in a humidified 5% CO_2_ atmosphere until they reached 70–80% confluence. Each experiment utilized a minimum of three distinct cell lines.

### 4.2. Reagents

β-tocotrienols, the monoclonal antibody against RhoA, PDGF-BB were purchased from Cayman Chemical (Ann Arbor, MI, USA), Santa Cruz Biotechnology (Dallas, TX, USA), and Wako (Osaka, Japan), respectively. In our previous study, it was observed that PDGF-BB at a concentration of 10 ng/mL induced ASM proliferation and migration. A dosage of 5–50 μM of γ-tocotrienol was used to assess the inhibitory effect on PDGF-BB-induced ASM proliferation and migration, and this effect was effectively reduced by the administration of 25 μM γ-tocotrienol [34]. Therefore, this dosage was selected for use in the current experiment.

### 4.3. Cell Proliferation Assay

Human ASM cells were seeded in 96-well plates at a density of 5.0 × 10^3^ cells/well in DMEM supplemented with 0.3% bovine serum albumin (BSA) and allowed to adhere for 24 h. Subsequently, the cells were treated with 5–25 μM β-tocotrienols for one hour, followed by incubation with 10 ng/mL PDGF-BB for 48 h. The Cell Counting Kit (CCK-8) (Dojindo, Kumamoto, Japan) was used to measure the proliferation of cells. A Sunrise microplate analyzer (TECAN, Mannedorf, Switzerland) was used to measure the results at 450 nm with a reference wavelength of 600 nm.

### 4.4. Cell Migration Assay

Cell migration was evaluated using a transwell chamber (Costar, Corning Incorporated, Corning, NY, USA). Human ASM cells were cultured in 100-mm dishes until nearly confluent, then maintained in a serum-free medium for 24 h. Following trypsinization, cells were resuspended in DMEM containing 0.3% BSA. After a one-hour treatment with 5–50 μM β-tocotrienols, 100 μL (5.0 × 10^4^ cells/well) of the suspension was added to the upper wells of the chamber, while 5 ng/mL PDGF-BB with 5–50 μM β-tocotrienols was placed in the bottom of the chambers. Following five hours of incubation, the cells were stained using Diff-Quick (Sysmex, Kobe, Japan) and then fixed with 10% formalin. Swabbing was employed to eliminate non-migrating cells from the top of the chambers. Migrated cells were counted manually in 10 randomly selected fields, and the average cell count per field was compared to that of the non-stimulated group.

### 4.5. Preparation of Cell Lysate and Western Blotting

Cells were rinsed with ice-cold phosphate-buffered saline (PBS) and subsequently lysed using a lysate buffer consisting of 500 μL 1× lysis buffer and 5 μL 100 mM PMSF. Equal amounts of protein were then subjected to electrophoresis and transferred onto polyvinylidene difluoride (PVDF) membranes (Amersham Hybond-P, GE Healthcare Lifescience, Buckinghamshire, UK). The membranes were incubated with primary rabbit antibodies: cyclin D1 (1:2000; Cell Signaling Technology); anti-rabbit phosphorylated and non-phosphorylated Akt1 (1:1000; Cell Signaling Technology, Danvers, MA, USA); polyclonal rabbit anti-phosphorylated or total antibodies for ERK1/2 (1:5000; Promega, Madison, WI, USA), followed by a second antibody incubation for 1 h at room temperature with horseradish peroxidase-conjugated anti-rabbit IgG (1:5000; GE Healthcare Lifescience). The bands were then visualized on an ImageQuant LAS 4000 mini (GE Healthcare Lifescience) using ECL reagent (GE Healthcare Lifescience) and densitometry quantified using TotalLab Quant software version 7.0 (Newcastle, UK).

### 4.6. Active Rho Detection Assay

Rho activity was measured according to the manufacturer’s instructions using an Active Rho detection kit (Cell Signaling Technology, Danvers, MA, USA). Human ASM cells were cultured in 100-mm dishes until they reached 60–70% confluence, followed by 24 h of maintenance in Ham’s F-12 (serum-free medium). The cells were then preincubated for one hour with 25 μM β-tocotrienols before being stimulated with 10 ng/mL PDGF-BB for five minutes. Subsequently, the cells were lysed in the lysis buffer (20 mM HEPES, 10 mM EGTA, 40 mM β-glycerophosphate, 1% Triton-X-100, 20 mM MgCl_2_, 2 mM Na_3_VO_4_, 1 mM DTT, 10 μg/mL leupeptin, 10 μg/mL aprotinin, 1 mM PMSF, 100 mM NaCl). To the cell lysate, 400 μg GST-Rhotekin-RBD was added and gently rocked for 1 h at 4 °C. Samples were run through gel electrophoresis and subsequently transferred onto PVDF membranes (Immobilon-P, Millipore, Billerica, MA, USA). Following this, the membranes were blocked with 5% non-fat dry milk in Tris-buffered saline (20 mM Tris, 150 mM NaCl, pH 7.6) containing 0.1% Tween 20 (TBS-T) for 1 h at room temperature. Then, the membranes were incubated overnight at 4 °C with 0.1% TBS-T containing primary mouse monoclonal IgG for RhoA (1:1000; Santa Cruz Biotechnology). After being washed with TBS-T, the membranes were incubated with a secondary antibody for 1 h at room temperature, using horseradish peroxidase-conjugated anti-mouse IgG antibody (1:3000). The bands were then visualized on an ImageQuant LAS 4000 mini (GE Healthcare Lifescience) using ECL reagent (GE Healthcare Lifescience) and densitometry quantified using TotalLab Quant software (Newcastle, UK).

### 4.7. Intracellular Reactive Oxygen Species Quantification

The level of ROS in ASM cells was measured utilizing the Oxiselect intracellular ROS assay kit (Cell Biolabs, San Diego, CA, USA) according to the manufacturer’s guidelines. The assay utilizes the cell-permeable fluorogenic probe 2′,7′-Dichlorodihydrofluorescin diacetate (DCFH-DA). Cellular esterases deacetylate DCFH-DA to the non-fluorescent 2′,7′-Dichlorodihydrofluorescin (DCFH), which is then rapidly oxidized to the highly fluorescent 2′,7′-Dichlorohihydrofluorescin (DCF) by ROS. In this investigation, serum-starved ASM cells were treated with 0.1 mM DCFH-DA for 1 h at 37 °C. Subsequently, human ASM cells were stimulated with PDGF-BB in the presence or absence of a 1-h pre-treatment with 25 μM β-tocotrienols. The intracellular ROS level was determined through fluorescence intensity, and quantification was carried out using a fluorescence plate reader (Infinite 500, TECAN).

### 4.8. Statistical Analysis

The data are presented as mean ± standard deviation (SD). Statistical differences between groups were analyzed using ANOVA. A subsequent multiple comparison test between pairs of groups was performed, with statistical significance defined as *p* < 0.05.

## 5. Conclusions

In conclusion, β-tocotrienols have demonstrated efficacy in reducing ASM cell proliferation and migration. We have also elucidated the key components of the downstream signaling cascade associated with the effects of β-tocotrienols on ASM cell proliferation and migration. This involves the inhibition of ROS production and RhoA inactivation. These findings suggest that β-tocotrienols may hold potential as a treatment for airway remodeling, which could benefit individuals with asthma. Further investigations are warranted to fully understand the potential of β-tocotrienols in addressing airway remodeling, especially through laboratory and clinical studies.

## Data Availability

Data is contained within the article.
